# Efficacy and safety of modified dual knife fistulotomy in complex ERCP cases involving type 3 papilla

**DOI:** 10.3389/fsurg.2025.1539012

**Published:** 2025-07-15

**Authors:** Qinkai Li, Mingjie Qian, Wei Cai, Zhenguo Qiao, Jianhong Zhu

**Affiliations:** ^1^ Department of Gastroenterology, The Second Affiliated Hospital of Soochow University, Suzhou, China; ^2^ Department of Gastroenterology, Suzhou Ninth People’s Hospital, Suzhou Ninth Hospital Affiliated to Soochow University, Suzhou, China

**Keywords:** ERCP, dual knife, type 3 papilla, fistulotomy technique, biliary cannulation

## Abstract

**Objectives:**

Cannulating protruding or pendulous type 3 papilla presents a higher level of difficulty in ERCP. Although pre-cutting papillotomy is a viable strategy, it is not without challenges. To overcome this issue, we developed a modified dual knife fistulotomy technique. The primary objective of this study was to assess the feasibility of a modified dual knife fistulotomy technique for achieving challenging biliary cannulation in type 3 papilla.

**Methods:**

This retrospective study investigated consecutive patients who underwent ERCP and dual knife fistulotomy for challenging biliary cannulation from 2017 to 2023. The study assessed the rates of technical success and adverse events related to dual knife fistulotomy during ERCP.

**Results:**

During the study period, a total of 380 patients with native type 3 papilla underwent ERCP. The initial success rate of biliary cannulation using the standard cannulation approach alone was 81.3% (309/380), which improved to 94.5% (359/380) by incorporating the double guidewire technique. Furthermore, the success rate further increased to 99.7% (379/380) with the addition of dual knife fistulotomy. Dual knife fistulotomy achieved a technical success rate of 95.2% (20/21) while no serious adverse events occurred.

**Conclusion:**

Dual knife fistulotomy is an effective technique for gaining access to the biliary system in cases of unsuccessful standard cannulation of type 3 papilla, with no significant increase in the risk of adverse events.

## Introduction

Successful bile duct cannulation is essential for performing therapeutic ERCP and subsequent interventions. However, failure to gain access to the bile duct is still a common problem. Precut sphincterotomy is used when conventional techniques fail to achieve selective biliary cannulation (1, 2). The technique significantly improves the success rate, but it is also associated with increased risks of adverse events such as bleeding and perforation. When the procedure is performed by qualified biliary endoscopists, early precut can reduce the risk of post-ERCP pancreatitis (3). The dual knife is commonly used for endoscopic submucosal dissection and has been reported to be safe and effective for precut papillotomy in difficult bile duct cannulation (4). Previous studies have demonstrated that the morphology of the major duodenal papilla affects bile duct cannulation, with type 2 small papilla and type 3 protruding or pendulous papilla posing greater challenges for cannulation compared to type 1 regular papilla (5). Type 3 protruding papilla facilitates the easier performance of fistulotomy due to its larger intraduodenal portion and “target” area. To overcome this issue, we developed a modified dual knife fistulotomy (DKF) technique (6). The primary objective of this study was to assess the feasibility of a modified DKF technique for achieving challenging biliary cannulation in type 3 papilla.

## Methods

### Patients

This retrospective study included consecutive patients aged 18 years or more with a native type 3 duodenal papilla who underwent ERCP and DKF for challenging biliary cannulation from 2017 to 2023. Papilla types were classified according to the criteria described by Haraldsson et al. (5). DKF was performed in cases of challenging biliary cannulation. Difficult biliary cannulation was defined by the presence of one or more of the following: more than 5 contacts with the papilla whilst attempting to cannulate; more than 5 min spent attempting to cannulate following visualization of the papilla; more than one unintended pancreatic duct cannulation or opacification (2). This retrospective study adhered to the principles outlined in the Declaration of Helsinki. Written informed consent was obtained from all patients for the ERCP and DKF procedures, and they were offered the option to opt out of having any information published.

### Technique

All dual knife fistulotomies were performed by a single experienced endoscopist (Dr. JHZ), who had performed over 1,500 ERCP and 800 ESD procedures including 20 precut cases with the needle knife. In cases of failed biliary cannulation, the double guidewire technique was employed as an option. If cannulation remained challenging despite the use of the double guidewire technique, DKF was performed using the dual knife, which was equipped with a 1.5-mm cutting knife (KD-650 Q; Olympus, Tokyo, Japan), in conjunction with a standard duodenoscope (TJF-260; Olympus, Tokyo, Japan). The generator (VIO 200S, ERBE, Tubingen, Germany) was set to ENDOCUT I, Effect 2, Duration 3, and Interval 3. The dual knife is one of the most constantly used knives in ESD and has several advantageous functions, including incision, dissection, and hemostasis. The KD-650Q is typically used for ESD of colorectal tumors with a knob-shaped tip measuring 1.5 mm in length. DKF was performed by retaining the indwelling pancreatic duct guidewire in patients who had previously undergone the double-guidewire technique. In our study, DKF was primarily performed in patients with prior pancreatic duct guidewire placement during the double-guidewire technique, in which the indwelling guidewire served as an anatomical landmark. In rare cases without pancreatic cannulation, DKF was not employed. Following fistulotomy, wire-guided biliary cannulation was attempted.

**Figure 1:**
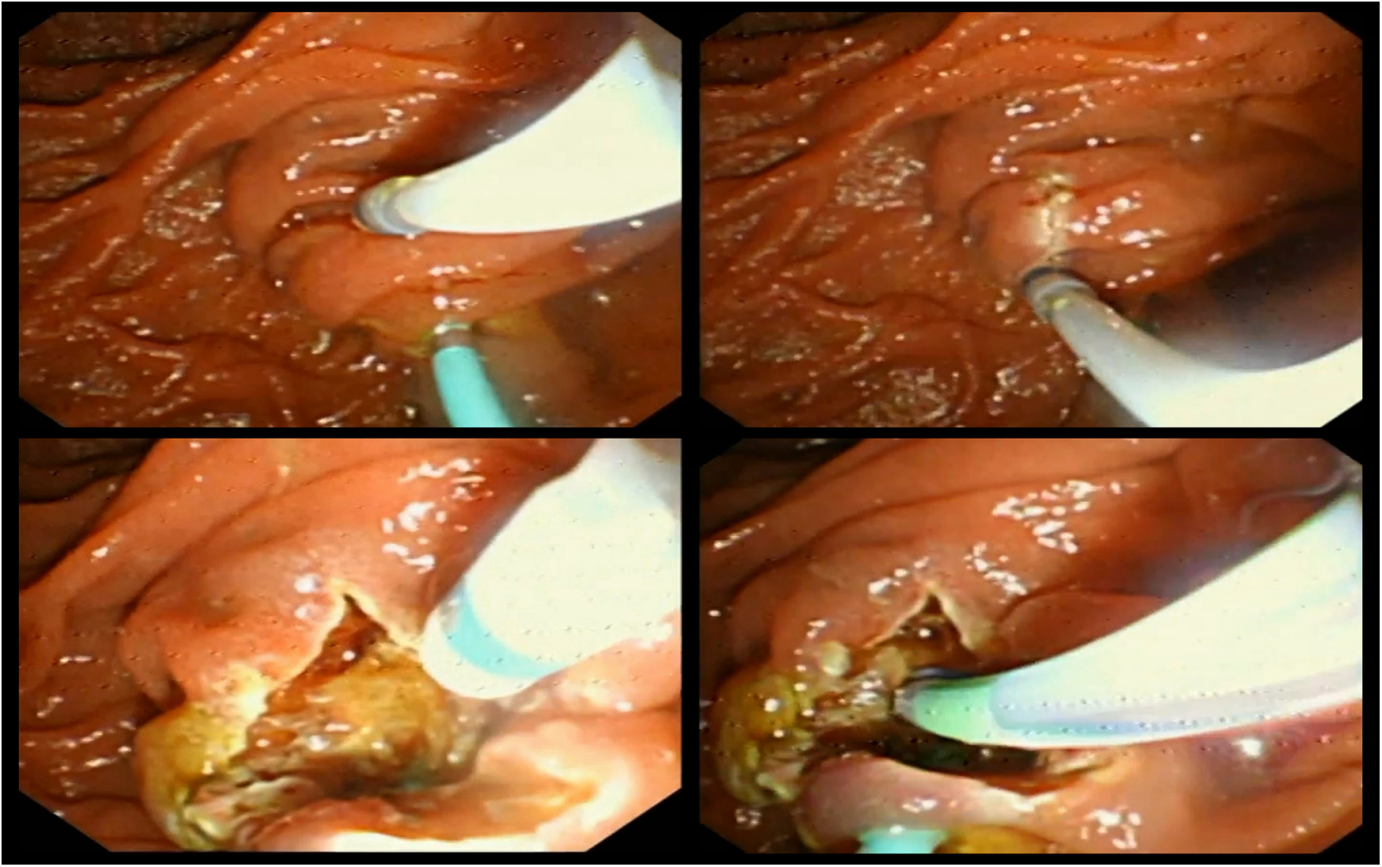
The operational procedure of dual knife fistulotomy.

In our previous report, we designed a modified DKF method aimed at facilitating mastery for beginners (6) (Figure 1).

The modified DKF technique used in this study aimed to enhance precision and safety, particularly for endoscopists with limited experience in precut procedures. The duodenal papilla was positioned slightly below and to the right of the center of the endoscopic visual field, while the dome of the papilla was placed centrally to optimize visualization. The incision was initiated at a site slightly above and to the right of the papillary dome. The knob-shaped tip of the dual knife was first inserted into the mucosa, followed by a controlled downward cut with the elevator of the forceps released, allowing for shallow and stable incision through the mucosal and submucosal layers. This approach preserved the native papillary orifice and maintained a consistent field of view throughout the procedure. The goal was to expose the underlying biliary bulge gradually, enabling identification of the bile duct trajectory with minimal risk of perforation or bleeding. Once the biliary bulge or the whitish submucosal bile duct wall became clearly visible, further incision was avoided, and wire-guided cannulation was initiated. This visual cue served as the key indicator for the adequacy of the fistulotomy. This technique was termed “modified” due to specific adjustments in the papilla positioning, incision angle, and visual guidance principles, aiming to improve orientation and minimize complications compared to conventional DKF. This modified technique was designed to provide better visual orientation, reduce technical complexity, and lower the risk of deep tissue injury compared to conventional needle-knife fistulotomy.

### Outcomes

The outcomes of this study were the technical success and adverse event rates associated with DKF. Technical success was defined as successful completion of the procedure, including sufficient fistulotomy, and biliary cannulation after fistulotomy. Adverse events such as bleeding, pancreatitis, and perforation, along with their respective severities, were determined based on established consensus criteria (7). Continuous variables such as operative duration were reported as median and range due to the small sample size and the presumed non-normal distribution.

## Results

**Figure 2:**
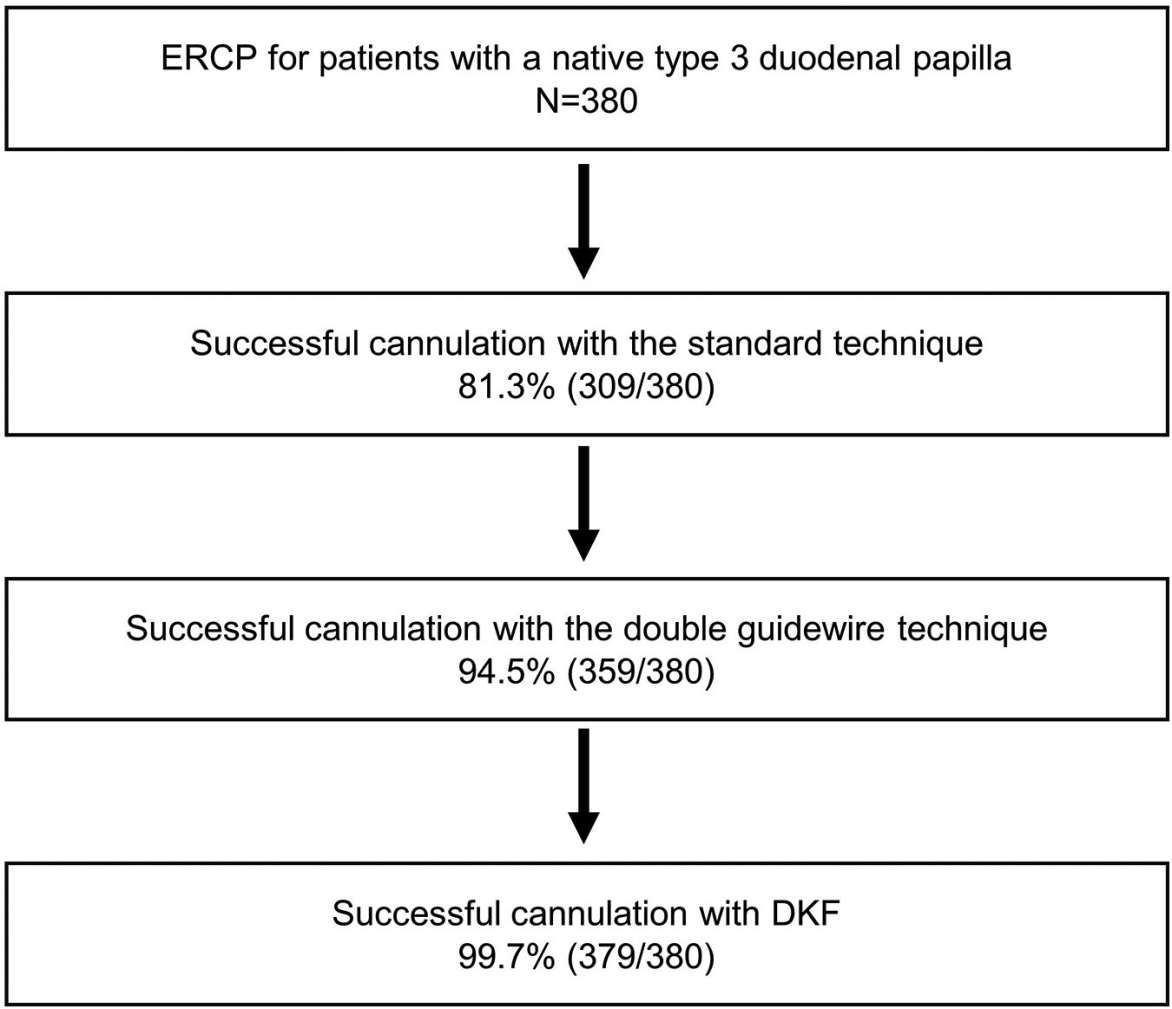
Flowchart detailing the outcomes of the patients included in this study.

During the study period, a total of 380 patients with native type 3 papilla underwent ERCP, and among them, 71 patients failed to achieve biliary access by the standard cannulation method. Among these 71 cases, successful biliary cannulation was achieved in 50 patients by employing the double guidewire technique. Subsequently, 21 patients underwent DKF and attained successful biliary cannulation, with only one case failing. The overall success rate of biliary cannulation using the standard approach alone was 81.3% (309/380). This success rate increased to 94.5% (359/380) when the double guidewire technique was incorporated in addition to precutting. Furthermore, with the additional implementation of DKF, the success rate further improved to 99.7% (379/380) (Figure 2). The characteristics and outcomes of the 21 patients in whom DKF was attempted are presented in Table 1.

**Table 1:** Baseline characteristics of the 21 patients and outcomes of DKF in ERCP.

Characteristic	Value
Sex, male/female	11/10
Median age, years	69 (29–93)
Indication for ERCP	
Choledocholithiasis	19 (90.5)
Malignant biliary stricture	1 (4.8)
Benign biliary stricture	1 (4.8)
Cholangitis	6 (28.6)
Jaundice	8 (38.1)
Urgent procedure	2 (9.5)
Periampullary diverticulum	4 (19.1)
Success of biliary cannulation after DKF	20/21
Median operative duration for DKF, min	5.5 (3.2–19.8)
Adverse events	0

The technical success rate associated with DKF was 95.2% (20/21). Only one patient, a 93-year-old elderly male admitted for choledocholithiasis, underwent a failed DKF procedure. The failure of DKF was attributed to the presence of extensive mucosal folds at the papilla, which posed challenges in identifying the bile duct following the mucosal incision. Although the failed case exhibited prominent mucosal folds, the papilla itself was consistent with type 3 morphology based on the classification proposed by Haraldsson et al. As a result, the patient underwent surgical intervention.

The median duration of the operation, from the start of DKF to successful cannulation, was 5.5 min (range: 3.2–19.8). All patients who underwent DKF did not experience any significant adverse events, such as postoperative pancreatitis, bleeding, or perforation.

## Discussion

According to the findings of this study, it is feasible to perform DKF in patients with difficult cannulation of type 3 papilla, and DKF improves the overall success rate of cannulation.

Precutting is an effective technique used in cases of difficult biliary cannulation when the standard technique has failed. Various precut techniques have been developed, including needle-knife papillotomy (NKP), needle-knife fistulotomy (NKF), and transpancreatic sphincterotomy (TPS) (8, 9). Precut papillotomy is a technically challenging procedure associated with a high incidence of adverse events, such as pancreatitis, bleeding, and perforations (10, 11). NKF involves making an incision in the papillary roof above the orifice without direct contact. Consequently, NKF can potentially reduce the occurrence of post-ERCP pancreatitis by preventing mechanical damage to the pancreatic orifice and minimizing injury to the pancreatic duct (12–14). According to the European Society of Gastrointestinal Endoscopy, needle-knife fistulotomy is recommended as the preferred technique for precutting (2).

Liu et al. first reported the application of the dual knife in the precut papillotomy for difficult bile duct cannulation (4). The dual knife in precut papillotomy for difficult bile duct cannulation is characterized by several advantages. Firstly, its knob-shaped tip and 1.5-mm length reduce the risk of slippage and deep tissue penetration during papilla cutting. The two-step knife extrusion allows for easy length adjustment without requiring confirmation under endoscopic view, addressing the limitations of the flex knife. Moreover, its smaller tip size compared to other knives enhances maneuverability during precut procedures. Secondly, the intumescent tip of the dual knife, combined with the coagulation current, effectively controls acute bleeding during the procedure. These features contribute to a high success rate, shorter procedure duration, and fewer adverse events.

We selected type 3 papillae for our study due to their protruding morphology. Although Lee et al. reported that the type of major papilla did not have a significant impact on the success rate, it is noteworthy that NKF demonstrated a high success rate for the protruding-type papilla (15). While direct cannulation presents increased challenges (5), employing a precut papillotomy facilitates the identification of the cutting plane, thereby enhancing the safety of the procedure.

These technical challenges in conventional precut techniques prompted the development of our modified DKF method, which was designed to improve visual orientation, incision stability, and safety, as detailed in the Methods section.

No adverse events were observed during the study. The incidence of bleeding, perforation, and pancreatitis was potentially lower compared to NKF.

The present study was constrained by its single-center design and small sample size, which limited its generalizability. The retrospective approach employed in the study introduced selection bias that could not be mitigated. Additionally, the majority of our patients presented with gallstone-related conditions, and the efficacy of the procedure in patients with other etiologies has yet to be established. All surgeries were performed by a single endoscopist who was proficient in both ERCP and ESD procedures. Although the modified DKF technique may simplify orientation and reduce the risk of adverse events compared to conventional precut methods, it still requires a baseline level of proficiency in advanced endoscopic handling, preferably including some experience with ESD instruments. These limitations indicate that the findings of this study may not be directly generalizable to other institutions. While the procedure demonstrates potential as a versatile technique, its feasibility must be confirmed through further multicenter analyses.

EUS-guided rendezvous was not performed in this case as interventional EUS techniques were not routinely available at our center during the study period. Given the patient's advanced age and comorbidities, surgical intervention was considered the most practical and definitive alternative.

A recent randomized controlled trial by Choudhury et al. compared endoscopic ultrasound-guided rendezvous (EUS-RV) and precut sphincterotomy as salvage techniques in patients with benign biliary disease and difficult biliary cannulation. The study demonstrated comparable technical success rates (92% vs. 90%), similar adverse event profiles (12% vs. 10%), and equivalent procedure durations between the two approaches (16). These findings suggest that EUS-RV may serve as an alternative salvage strategy in expert centers. However, EUS-RV requires advanced equipment, operator expertise, and may not be readily available in all settings. In contrast, modified DKF can be performed using standard ERCP tools, potentially offering a more accessible and practical salvage option in institutions without interventional EUS capacity.

DKF is a highly effective technique for achieving biliary system access in cases where standard cannulation of a type 3 papilla is unsuccessful, without a notable increase in the risk of adverse events.

## Data Availability

The raw data supporting the conclusions of this article will be made available by the authors, without undue reservation.
